# High-resolution coupled physics solvers for analysing fine-scale nuclear reactor design problems

**DOI:** 10.1098/rsta.2013.0381

**Published:** 2014-08-06

**Authors:** Vijay S. Mahadevan, Elia Merzari, Timothy Tautges, Rajeev Jain, Aleksandr Obabko, Michael Smith, Paul Fischer

**Affiliations:** Argonne National Laboratory, 9700 S. Cass Avenue, Argonne, IL 60439, USA

**Keywords:** multi-physics, reactor analysis, code coupling

## Abstract

An integrated multi-physics simulation capability for the design and analysis of current and future nuclear reactor models is being investigated, to tightly couple neutron transport and thermal-hydraulics physics under the SHARP framework. Over several years, high-fidelity, validated mono-physics solvers with proven scalability on petascale architectures have been developed independently. Based on a unified component-based architecture, these existing codes can be coupled with a mesh-data backplane and a flexible coupling-strategy-based driver suite to produce a viable tool for analysts. The goal of the SHARP framework is to perform fully resolved coupled physics analysis of a reactor on heterogeneous geometry, in order to reduce the overall numerical uncertainty while leveraging available computational resources. The coupling methodology and software interfaces of the framework are presented, along with verification studies on two representative fast sodium-cooled reactor demonstration problems to prove the usability of the SHARP framework.

## 1. Introduction

High-fidelity computer simulations of multi-physics problems require solving large systems of complex, coupled, nonlinear, stiff equations. Many examples of nonlinear multi-physics phenomena occur in a spectrum of scientific fields, raising the need to develop and validate accurate and stable numerical modelling and solution procedures. Such robust coupling methods are often used in radiation hydrodynamics, nuclear reactor analysis, fluid–structure interaction and climate model problems because of the need to accurately resolve the fine-scale effects in physics evolution [1].

Traditional solution techniques for coupled multi-physics phenomena have often relied on operator-split (OS) coupling strategies, which can introduce several sources of numerical errors in the solution fields as a result of inconsistent spatio-temporal treatment of the nonlinear terms. It is imperative to verify the accuracy preservation in these methods for problems of interest since effective resolution of the disparate characteristic physical scales is non-trivial. In this paper, we introduce an integrated multi-physics coupling capability with a multi-resolution hierarchy that is designed to ultimately span the full range of length and time scales present in relevant nuclear reactor design and safety analyses.

In order to produce a flexible multi-physics simulation capability, two obvious approaches can be pursued. In one approach, pieces of existing single-physics codes can be assembled into an overall coupled simulation code with appropriate interfaces to communicate between the components. This is generally referred to as a ‘bottom-up’ framework approach (MCT [2], SHARP [3,4]). The other approach is to use an integrated, coupled-physics modelling framework, with new code pieces for each relevant physics area developed inside that framework. This is sometimes referred to as a ‘top-down’ framework approach (DUNE [5], MOOSE [6], KARMA [7], among several others). The former approach takes advantage of the fact that several man-years invested in these existing verified and validated individual physics modelling codes are reusable, but producing a multi-physics capability will then require some intrusive modifications to enable appropriate software interfaces. The top-down framework approach avoids such intrusive implementations by providing unified physics interface guidelines that simplify the software overhead but at the substantial cost of re-writing all the necessary physics models from scratch, including their verification and validation (V&V) suites.

The implementation of a verified multi-physics solver code also imposes a number of requirements on the overall design aspects of the framework. Hence we need flexible interfaces and robust solver options that encompass variations in a hierarchy of coupling algorithms affecting the frequency and degree of coupling between the physics. Since the choice of a coupling method is both physics and problem-specific, SHARP includes a spectrum of numerical techniques to tackle the relevant scales in physical phenomena that is relevant to nuclear reactor design. The necessary background on the methods is provided in the following sections.

### (a) Background

For illustration, let the nonlinear vector-valued function representing a coupled partial differential equation (PDE) system be written in a general form as
1.1
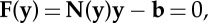

where **y** is the solution vector that is dependent on both space and time, respectively, and 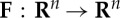, where **F** is the nonlinear operator representing the coupled system and *n* is the total number of unknowns. For ease of comprehension, we can write **F** as in the second equality of equation (1.1), where **N** is also a nonlinear operator and **b** is the load vector. It helps to represent **y** as a vector comprised of the solution vector for each of the *M* physics components involved, i.e. [**y**_1_,**y**_2_,…,**y**_*M*_]^T^. A similar definition holds for **F**(**y**), and its *m*th component is the nonlinear residual stemming from the *m*th physics component and may depend effectively on all other fields, for example, **F**_*m*_(**y**)=**F**_*m*_(**y**_1_,**y**_2_,…,**y**_*M*_).

### (b) Explicit coupling strategies

**Figure 1.**
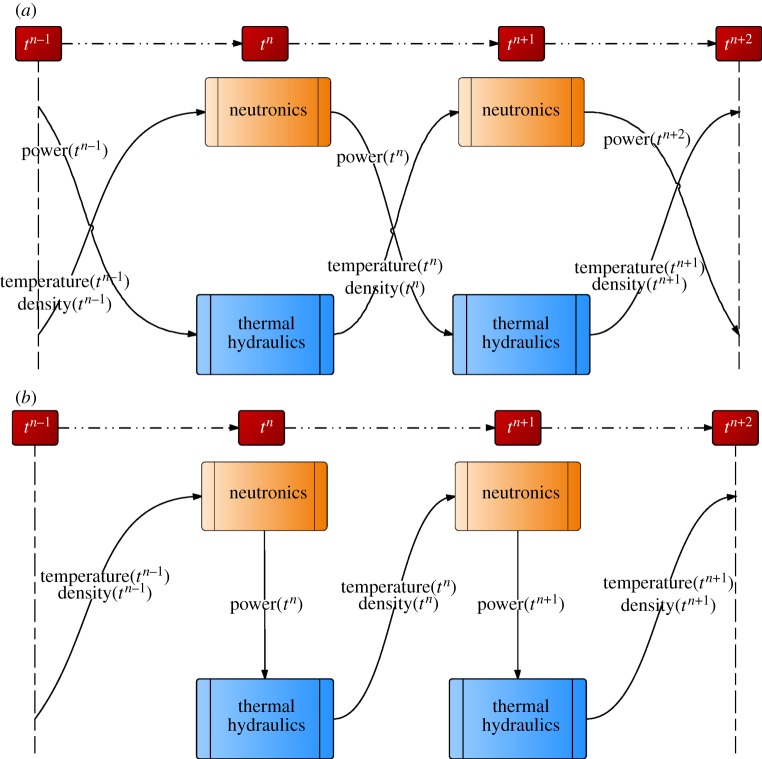
Two lower order OS coupling strategies. (*a*) Simultaneous OS coupling and (*b*) staggered OS coupling. (Online version in colour.)

In the past few decades, high-fidelity modelling of nonlinear multi-physics problems has been subdivided into several distinct domains of physics and solved individually as mono-disciplinary blocks with specialized codes, without rigorous coupling between the different physics using OS. With the advent of parallel virtual machines and the message passing interfaces (MPI) in the 1990s, the OS coupling of several existing specialized single physics codes has become the main multi-physics paradigm in reactor analysis. This kind of modelling is based on coupling several existing specialized mono-disciplinary codes using a ‘black-box’ strategy, where the input of one code is the output of other, thereby producing solutions that are weakly coupled. Schematics of such models are shown in figure 1, where the system of PDEs arising from the spatial and temporal discretization of physical models is decomposed into simpler subproblems. Each physics component is solved by an independent, specialized single-physics code and the data between codes are exchanged through message passing paradigms. Often, this strategy is non-iterative, and the nonlinearities due to the coupling between the physics components are not resolved over a time step, reducing the overall accuracy in the time-stepping procedure to first order *O*(Δ*t*), even though high-order time integration might have been used for the individual physics components [8,9]. Note that this explicit linearization of the problem in the OS strategy does not resolve the nonlinearities between the different physics. Yet, these isolated physical models in reality describe physical phenomena that are tightly intertwined and rely heavily on the solution field of each other.

For illustration, consider the nonlinear coupled system shown in equation (1.1). In the OS loose coupling strategy, the nonlinear operator is linearized as follows through an explicit treatment:
1.2
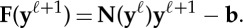

Hence the new update to the solution is obtained by solving the system
1.3
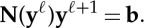

Although OS allows parts of the problem to be treated implicitly and others explicitly, the lack of iterations in the conventional strategy degrades the solution accuracy in time to first order, and the explicit linearization imposes a conditional stability limit for the time-step selection. The direct implication of using smaller time steps to achieve a reasonable accuracy is that the computations need greater CPU time and resources. Despite these drawbacks, this is still one of the major coupling paradigms used today for solving nonlinear multi-physics systems.

The attractive feature of such a coupling strategy is that the legacy of many man-years of mono-disciplinary code development and V&V is preserved. It is of prime importance to analyse the coupling strategies that can produce highly accurate solutions even in the complex scenarios usually encountered in multi-physics applications. As mentioned earlier, nuclear reactor analysis is a good example of a highly nonlinear, coupled, stiff problem, and the nonlinearities at the heart of reactor design, analysis and safety calculations provide a good state-space to test robust, high-fidelity numerical methods for multi-physics problems. Physical phenomena such as those found in reactor accidents involve rapidly varying transients yielding stiff systems of differential equations that are characterized by solutions having fast varying modes together with slower varying modes, requiring time integrators that can handle such disparate time scales. Stiff problems necessitate the use of implicit time discretization for stability reasons, indicating that non-iterative OS coupling could prove disadvantageous in terms of efficiency (cost for obtaining a certain accuracy in the solution).

Current examples of multi-physics coupling in the field of nuclear reactor analysis involve the following pairs of deterministic neutronics/thermal-hydraulics codes: NURESIM based on SALOME [10], PARCS/TRACE [11] and NESTLE/RELAP [12] and other variations with stochastic neutronics methods with MCNP/Star-CCM [13,14]. More recently, research on using OpenFOAM for performing fine-scale modelling of pressurized water reactor cores [15] has also been explored with moderate success. Several advanced OS strategies exist that can yield up to second-order accurate solutions in time, but they are complicated to implement generally in the context of legacy codes and hence are not typically employed. For more details regarding these higher order OS schemes, we refer the reader to [16–19]. In this paper, we perform successively iterated OS schemes that are fully converged via Picard iterations so that the nonlinearities are resolved at every time step thereby yielding unconditional stability and recovering high-order accuracy. The linear rate of convergence is accelerated with the Steffensen method and with an explicit second-order predictor [9].

### (c) Implicit coupling strategies

An alternative to explicit or loosely coupled OS strategies is to converge the nonlinearities between the physics at every time level to obtain a tightly coupled solution that is consistent with the nonlinear system of PDEs. This preserves the higher order temporal accuracy of specialized schemes that can be used to resolve the disparate temporal scales in the different physics. Even though the cost/(time step) can be larger than that of an OS time step, we stress that the stability of the higher order discretization scheme can be maintained by using this procedure, unlike the explicit linearization (OS) method where the solution is only conditionally stable.

To devise such a tightly coupled solution procedure, one needs to apply a nonlinear iterative scheme in order to solve the coupled physics and converge the dependent terms to within user-specified tolerances. Two techniques for nonlinear system of equations are mentioned next: the Picard iteration technique (or variations of nonlinear Richardson) and the well-known variants of Newton's method via Jacobian-free Newton–Krylov (JFNK) approximations [20]. In this paper, we will concentrate on the former, because it allows effective reuse of existing physics components directly and avoids solving large monolithic systems by solving linearized subproblems in a scalable fashion, thereby reducing the overall computational complexity of the simulation.

**Figure 2.**
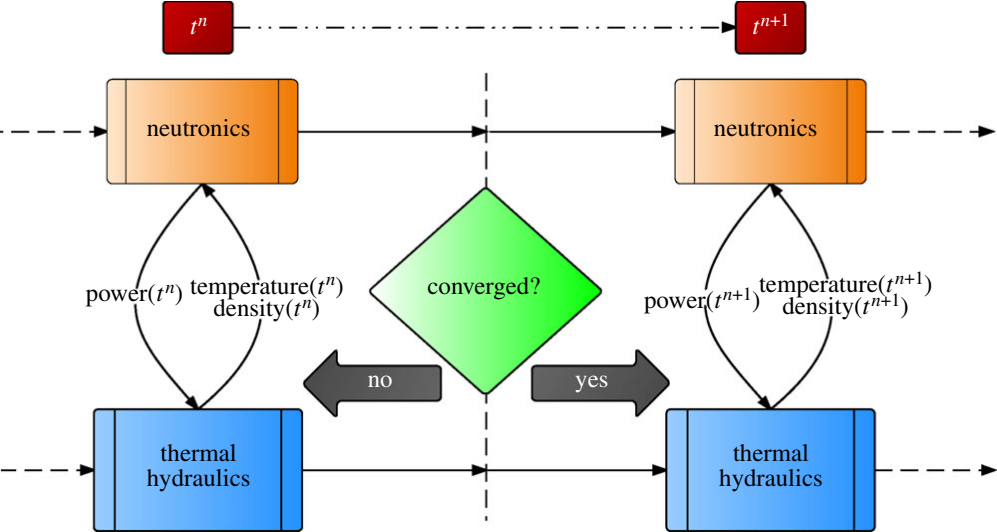
Higher order, converged, iterative split coupling strategy. (Online version in colour.)

The Picard iteration technique is a well-analysed nonlinear method that can be used to converge the nonlinearities over the different physics when an OS coupling technique is employed to couple multiple physics codes. Picard iterations can restore the convergence order of a higher order scheme and eliminate the loss of accuracy due to the crude explicit linearization in a loosely coupled strategy. The schematic for such a method is shown in figure 2. This essentially involves iterating over the solution obtained by successively solving equation (1.3).

The advantage of such a coupling scheme is that it is non-intrusive and can easily use an existing framework of codes to obtain a tightly coupled solution, an approach rightly suited for SHARP. But the primary disadvantage of using such a strategy to restore the accuracy is the increase in computational cost due to linear convergence of Picard iterations. In order to overcome this issue, some form of nonlinear acceleration technique is necessary to make this scheme efficient and feasible [9]. Previous research using Aitken's iterated Δ^2^ technique suggests that usage of such acceleration schemes can be advantageous, and efforts to apply the Wynn epsilon method [21] and other schemes should be pursued as future extensions.

An example discrete coupled system of equations to be solved is of the following form:
1.4
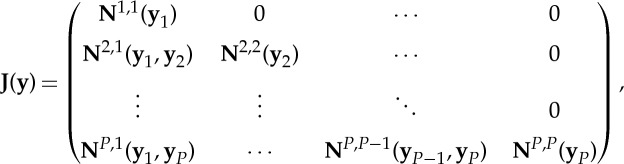

where *i*,*j*∈[1,*P*] with *P* being the total number of coupled physics components, **N**^*i*,*j*^ represent the nonlinear block corresponding to physics *i* coupled to solution from physics *j* and **y**_*i*_ is the *i*th physics component solution.

Since the diagonal nonlinear blocks **N**^*i*,*i*^(**y**_*i*_)∀*i*∈[1,*P*] require only the inversion of the single physics operator, this fixed point iteration procedure can be continued to generate a sequence of solutions that converge to the true coupled physics fields. Equation (1.4) produces a global nonlinear Richardson iteration procedure with a Jacobian matrix that resembles Block–Gauss–Siedel (BGS) coupling between the physics and hence can be solved effectively by solving sub-problems using existing mono-physics codes. Note that in this general framework, several different block structured matrices can be used to capture stronger physics coupling between the components, instead of the BGS scheme shown in equation (1.4).

In this paper, we investigate the bottom-up approach for performing coupled multi-physics analysis of reactor core systems using the SHARP framework with a flexible options-based implementation to test both loosely coupled and fully converged (Picard) coupling strategies. The organization of the paper is as follows. In §2, a brief description of the components of the SHARP framework is provided along with some implementation details for the interfaces. Then we show results in §3 on verification studies performed on a fully heterogeneous assembly-scale problem in order to gauge efficiency and accuracy metrics that give the necessary foundation for simulating a large-scale, realistic nuclear engineering benchmark validation problem.

## 2. SHARP: a coupled multi-physics simulation toolbox

One can construct a multi-physics reactor core modelling code in many ways, and numerous efforts have attempted to do so by providing a stepping stone for future efforts [1]. What distinguishes the SHARP effort from others is the goal of flexibility in the physics model and implementations, underlying discretization types, multi-fidelity resolution and flexible software options supported by the framework. We begin by describing the components that make up the SHARP coupled multi-physics code framework, and we then describe necessary modifications to integrate existing physics components into this framework.

As stated in §1, the ‘bottom-up’ approach lends itself naturally to leverage existing well verified libraries such as the Mesh-Oriented datABase (MOAB [22]), for handling and manipulating the discrete mesh representation, the coupled physics driver, Coupled Physics Environment (CouPE), which is built on a state-of-art scalable solver library (PETSc [23]) to provide an array of coupling strategies, controllable with command-line options (no recompilation). Using an existing physics code in this system requires that the system support whichever mesh type(s) the individual physics natively uses. The physics models can retain their own native representation of the discrete mesh, which gets transferred to and from MOAB's representation through a mesh adaptor or alternatively, it can use MOAB internal representation directly through the language interoperable interfaces.

In practice, the coupled system may be solved on multiple physics meshes, each of which models part or the entire physical domain of the problem but resolving relevant spatial scales pertaining to a single physics. In order to perform efficient coupled calculations, the results must be transferred from the physics/mesh on which they are generated (source) to the physics/mesh for which they provide initial or boundary conditions (target) due to nonlinearity introduced by physics coupling. ‘Two-way’ transfer is required in cases where the physics depend on each other's solution fields, for example in reactor analysis where neutronics computes total fission heat generation based on material properties that are temperature dependent, which are computed by thermal-hydraulics component using the heat generation source term computed by neutronics.

**Figure 3.**
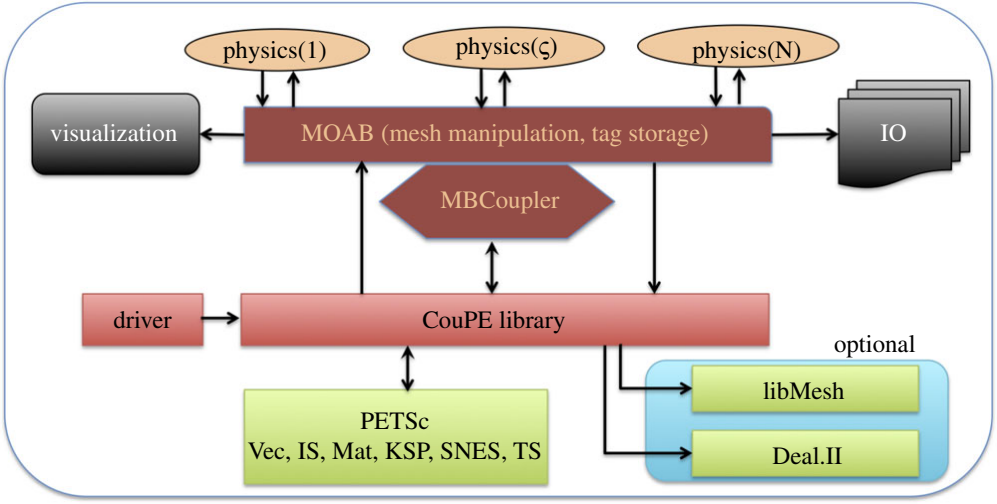
Depiction of the SHARP framework, with MOAB as data backplane and CouPE driving the standalone or coupled physics calculations. (Online version in colour.)

Figure 3 illustrates the schematic of the SHARP framework used in this paper. MOAB provides a representation of the meshes, and MBCoupler (a MOAB based tool for parallel solution data transfer [24]) to interpolate (or via *L*_2_ projection) each dependent physics component solution from the source to the target, with appropriate conservation prescriptions [25]. The CouPE library is responsible for implementing multi-physics coupling methods to consistently and accurately couple the different components, in order to solve the nonlinear reactor-physics problem. The combination of these tools provides the basis for the SHARP component- based framework.

### (a) Mesh-Oriented datABase

MOAB is a library for query and modification of structured and unstructured mesh and field data associated with the mesh [22]. MOAB can represent all entities typically found in the finite-element (FE) zoo, as well as polygons, polyhedra and structured meshes. MOAB provides parallel functionality for resolving entity sharing and ghosting between processors, with sharing and ghosting information available as annotations on the local mesh [26].

The data model in MOAB consists of four fundamental data types:

— *Entity*: basic entity in the discrete model, e.g. vertex, quadrilateral and tetrahedron.
— *Entity set*: arbitrary collection of entities and other sets.
— *Interface*: database object or instance from an implementation point of view.
— *Tag*: set of data annotated on objects of any of the other three data types.

This data model, while simple, is flexible enough to represent the necessary data to run coupled physics simulations. In particular, data tags can be used to store both fine-grained solution data on individual vertices and elements and coarse-grained annotation of sets to identify them as boundary conditions, material types or processor partitions. With such a uniform mechanism to push and pull data through the MOAB mesh layer, a clear physics abstraction can be created to transfer the solution from a source physics mesh to a target physics mesh, to be used as a source term or to evaluate dependent parameters.

One of the critical aspects in assembling a multi-physics modelling code is mapping the results from one physics domain to another. We use MOAB as a ‘data backplane’ to link physics through their spatial domains; and we use MBCoupler to project coupled physics fields between those domains. MBCoupler has been demonstrated to be accurate and was recently shown to have more than 65% strong scaling up to 262 000 processors on the BG/Q (Mira at Argonne). This tool allows the source and target meshes to be partitioned and distributed across processors in a way that is performance optimized for the individual physics associated with the mesh. Target-to-source mesh point location is performed in parallel, with bounding box-based acceleration used to determine possible source mesh processors containing every point and with KD-tree (or optionally BVH-tree) decomposition used locally on each processor. MBCoupler can transfer solutions using piecewise linear and quadratic FE shape functions along with options to use spectral element (SE) shape functions depending on physics discretizations. In this paper, MBCoupler is used to map the results computed by one physics module onto the discrete mesh used by another, in order to evaluate the nonlinear terms in the tightly coupled, successive iterative splitting scheme.

In the SHARP framework, we have implemented MOAB interfaces to several different physics components that are relevant to fast reactor physics analysis.

### (b) CouPE

CouPE aims to enable scalable and extensible coupling of different physics components that are nonlinearly dependent on each other. The SHARP multi-physics framework for solving reactor analysis problems employs validated and verified efficient mono-physics codes with MPI-level parallelism to implement several coupling strategies including OS and tightly coupled, iterated methods. The current design of CouPE is intended to satisfy the need for a loosely coupled software framework even when the physical phenomena are strongly coupled to each other.

The motivations in designing CouPE are the following:

(i) Use existing libraries and physics codes in order to minimize development time and leverage man-years invested in these tested codes.
(ii) Enable a flexible and accurate data exchange framework between codes in a mesh-, numerics- and physics-aware fashion; in other words, data exchange while maintaining consistency, accuracy and conservation of key fields that contribute to inaccuracy and stability issues in coupling.
(iii) Provide flexible data containers and physics objects that facilitate and simplify the evaluation of the nonlinear residuals representing the fully discrete PDE for different physics components.
(iv) Provide the ability to use different kinds of multi-physics coupling strategies within the same architecture with runtime object polymorphism, avoiding recompilation for each problem.

CouPE aims to integrate all the physics components under a unified framework in order to exchange the solution from one physics to another (interfaces to MBCoupler) and converge the coupled physics solution fields to user-specified tolerances (typically 10^−4^ – 10^−8^ [27]) without sacrificing numerical stability or accuracy. CouPE provides the necessary components and layers to wrap existing physics codes or write a complete description of a physics problem from scratch in order to solve phenomena of interest, that is, to enable both bottom-up and top-down approaches. The library provides the necessary tools to quickly implement any of the popular variations of an OS coupled solver (Marchuk, Strang and Yanenko among others) or a more rigorous matrix-free inexact-Newton solver with a JFNK technique [20].

Similar to the PETSc library, CouPE is designed to allow the user to specify command-line arguments in order to control the dynamic behaviour of the coupled solver. The parameter specifications include the input for individual physics components, input mesh parameters, and type of solver along with ability to enable dynamically choosing the fidelity of the physics being coupled. This is made possible by abstracting out behaviour of the core object until runtime. The advantage of such a method is that the implementation of the coupled physics driver and the accompanying physics components need to be compiled, linked and verified only once and then can be re-used in a variety of different coupling methods (e.g. loose versus tight coupling). The coupled fields are iterated until convergence to user-required accuracy for any problem of interest. Results obtained by using this coupled physics driver based on the CouPE library are shown in §3.

### (c) PROTEUS

PROTEUS is a high-fidelity deterministic neutron transport code [28] (formerly UNIC), based on the standard second-order even-parity formulation [29] which is obtained from reformulation of the multigroup Boltzmann transport equation in the steady-state (SS) form shown below:
2.1
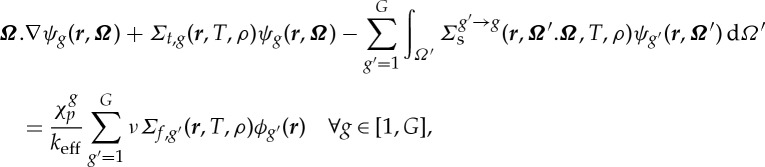

where *ψ*_*g*_(***r***,***Ω***) and *ϕ*_*g*_(***r***) are the angular and scalar flux for energy group *g*, respectively, *Σ*_*t*,*g*_ is the total material cross section in energy group *g* at position ***r***, 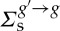 is the differential scattering cross section representing the probability that a particle at ***r*** in energy group *g*^′^ travelling in the direction ***Ω***^′^ is scattered into energy group *g* with direction d***Ω*** about ***Ω***, and 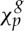 is the fission spectrum.

Expressing the angular flux in its even (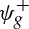) and odd (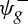) parity components, we have
2.2
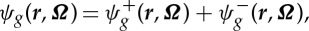

where
2.3
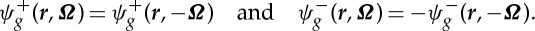



The final form of the even-parity equation is obtained by substituting the definitions equation (2.2) and equation (2.3) into equation (2.1) and algebraically manipulating the resulting equations to eliminate the odd-parity flux [29,30]. After applying continuous FE spatial discretization and discrete ordinates treatment of the angular terms, the discrete form of equation (2.1) and the even-parity equation resemble a generalized eigenvalue problem. The fundamental (dominant) eigenpair, i.e. eigenvalue *k*_eff_ and its corresponding eigenmode *ϕ*(*r*), provides critical information for the design of nuclear reactors. Since the scalar flux solution is obtained as a solution of the eigenproblem, only the shape of the flux can be ascertained and the magnitude is determined based on the total power load chosen during operation.

A nuclear reactor core is typically composed of hundreds of different materials and isotopes, each with different cross sections. The cross section of the material in equation (2.1) is greatly affected by the temperature and density of the material and depends on the energy of the incident neutron. In fully heterogeneous simulation models, the material cross sections are pre-processed and collapsed to the number of energy groups as required for the simulation by solving several fine-scale problems in different configurations so as to preserve the net reaction rate in the core. These cross sections are usually also tabulated, or provided in a closed form approximation, as a function of fuel and coolant temperatures (extension to additional parameters, such as boron concentration, void history, control rod history, etc., is straightforward). The tabulated cross-section values are obtained using table look-up and **R**^*p*^ interpolation, where *p* is the total number of parameters used. In this paper, we use the effective cross-section generation capability provided by *MC*^2^-3 code [31] that can collapse ultrafine group resolution of the ENDF data to multi-group data usable for problems of interest.

PROTEUS has a hierarchical multilevel solver based on space–angle–energy parallelism which internally uses PETSc's Krylov methods and SSOR preconditioners. This solver is capable of using several existing petascale parallel machines with demonstrated scalability of over 70% (strong scaling) at over 250 000 processors (BlueGene/P [32]). Recent implementations have also focused on enabling a two-grid multigrid preconditioning technique [33] to accelerate the solver. Written primarily in Fortran90, the interfaces to MOAB enable in-memory parallel mesh manipulation and transparent solution access from thermal-hydraulic and structural mechanic physics to account for the nonlinear feedback effects. The transients simulated in this paper use a quasi-static time-dependent capability [29] that requires recomputing the eigenproblem for the flux shape, while using point reactor kinetics equations to update the evolution of the total power in the reactor.

### (d) Nek5000

The Nek5000 computational fluid dynamics solver is based on the SE method developed by Patera [34]. For the underlying equations in the solver, we consider the conservation laws assuming incompressibility constraints and a constant fluid density. This assumption is a good approximation for the liquid sodium being modelled in the demonstration problems, and can be easily extended to mildly compressible flows (*M*<0.3). Then, the conservation of mass, momentum and energy equations are written as
2.4
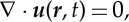

2.5
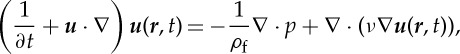

2.6
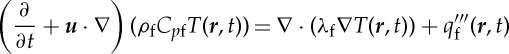

2.7
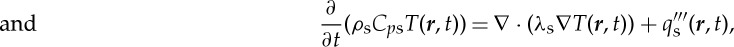

where ***u*** is the velocity field, ***r*** are the spatial coordinates, *t* is the time, *ν* is the kinematic viscosity, *C*_*p*_ is the heat capacity, *T* is the temperature, λ is the conductivity, *p* is the pressure, *ρ* is the density and *q*^′′′^_s_ and 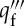 are the volumetric heat generation rates in the solid and fluid, respectively. The subscripts f and s refer, respectively, to the coolant and the solid components.

The open-source Nek5000 code supports two different formulations for spatial and temporal discretization of the incompressible Navier–Stokes equations: the *P*_*N*_−*P*_*N*−2_ method [35–37] and *P*_*N*_−*P*_*N*_ formulation [38,39]. Both formulations yield a decoupled set of elliptic problems to be solved at each time step including a Poisson equation for the pressure. For marginally resolved large-eddy simulation (LES) cases, we find that the higher order pressure approximation of the *P*_*N*_−*P*_*N*_ methodology tends to yield improved solution estimates, and this is consequently the formulation used for the calculations performed here.

Time integration is performed using a backward differentiation/extrapolation scheme of the second order (BDF2/EXT2) using variable time stepping. The Courant number is maintained below 0.5, with time steps in the range 10^−3^ s to 10^−6^ s. The Poisson equation for the pressure, the most computationally expensive stage, is solved using an AMG preconditioner. Nek5000 does not rely on external linear algebra packages. Nek5000 is massively parallel and employs the MPI standard for parallelism.

Typically, the solution of thermal-hydraulic modelling of reactor cores involves the solution of a modified conjugate heat transfer problem in rod bundles (fuel assemblies). The heat generated in cylindrical pins containing the nuclear fuels is removed by an external coolant flowing in parallel to the pins. In addition to the equation described by (2.7), additional transport equation may be solved to introduce turbulence modelling.

With a solution to equation (2.1), the volumetric heat generation rate *q*_s_′′′ estimated by equation (2.8) is used as the primary source term in the energy balance equation
2.8
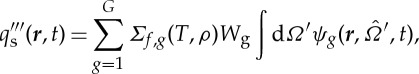

where *Σ*_*f*,*g*_(*T*,*ρ*) is the temperature- and density-dependent fission cross section of the material, *W*_g_ is the group-dependent power production per fission reaction and *ψ*_*g*_ is the angular flux for energy group *g*.

The boundary conditions for the described conjugate heat transfer problem in rod bundles are standard and are applied at the inflow, outflow and wall surfaces as follows:

— The inlet fluid surface has uniform prescribed velocity and fixed temperature.
— The inlet and outlet solid surfaces of the duct have an adiabatic temperature boundary condition.
— Standard outflow boundary conditions are specified at the outlet fluid surface.
— The wall surfaces of the pins and the inner surfaces of the duct have a non-slip velocity boundary condition.
— The outer surfaces of the duct have an adiabatic temperature boundary condition.

### (e) Implementation notes

At the most basic level of capability, integration of a given physics code into this system requires reading the mesh along with the associated data and writing solution fields back onto the mesh after their computation. This enables the interfaces in CouPE to transparently interface with the physics solvers and to pull/push the necessary data to drive the global nonlinear solver to convergence.

The convergence criteria for the solvers are determined based on the actual *L*_2_ global error of the numerical solution *U*_num_ computed on the corresponding physics mesh using the following definition:
2.9
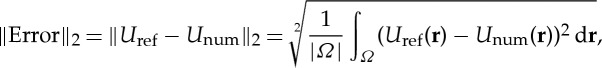

where *Ω* is the spatial domain and *U*_ref_ is the reference solution computed on a refined/resolved spatio-temporal grid.

## 3. Results

Verification of single-physics codes is a daunting task, and implementing the process for a complex multi-physics simulation requires quantifying errors at many stages. The spatial projection errors that occur when the solution field is transferred from one physics to another dominate the coupling errors along with the treatment of the nonlinear terms. In this paper, we present two demonstration problems that will help determine the accuracy of the solvers. First, a simplified hexagonal assembly (SAHEX) is taken and we test the data transfer mechanism for optimal accuracy and consistency as the source and target meshes are refined. Then, a realistic assembly that was employed in the EBR II reactor (the instrumented XX09 assembly) is solved for various SS and quasi-static transient problems, in order to investigate the advantage of using tightly coupled solvers to numerically resolve the relevant scales in the problem. The boundary conditions for the problems described are summarized in §2.

### (a) Initial conditions and transient specification

The neutronics solution without any coupled feedback is essentially a linear generalized eigenvalue problem that computes the fundamental eigenvalue, eigenmode pair. The neutronics solver assumes constant material-dependent temperature/density values to initiate computation of cross sections which are nominal values obtained with the initial conditions (ICs) from thermal-hydaulic solver. Owing to the nonlinear nature of the coupled physics, the hydraulics solver assumes an axially cosine shape as the heat source to compute the thermodynamically equilibrium solution to a user specified tolerance.

Each problem was tested in a two stage process:

— compute coupled initial SS solutions at rated conditions and
— perform a quasi-static transient where the power, temperature and density evolve based on the change in total reactivity.

The type of transient examined in the paper is a simplified loss-of-heat-sink, where the temperature of the fluid at the inlet boundary is specified as a function of time and the evolution in the coupled fields is computed. This simulates an accident scenario when the heat exchangers fail to remove excess heat from the coolant, thereby increasing the inlet temperatures steadily, causing feedback effects from different sources to interact nonlinearly between the physics:
3.1
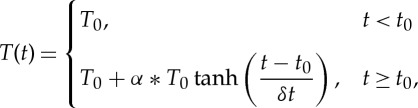

where *t*_0_ is the transient initiation time, *T*_0_ is the initial converged temperature solution, *δt* is the duration of the transient at the inlet and *α* is the damping parameter to control the magnitude of the perturbation (typically 0.2).

### (b) Simplified hexagonal fuel assembly

**Figure 4.**
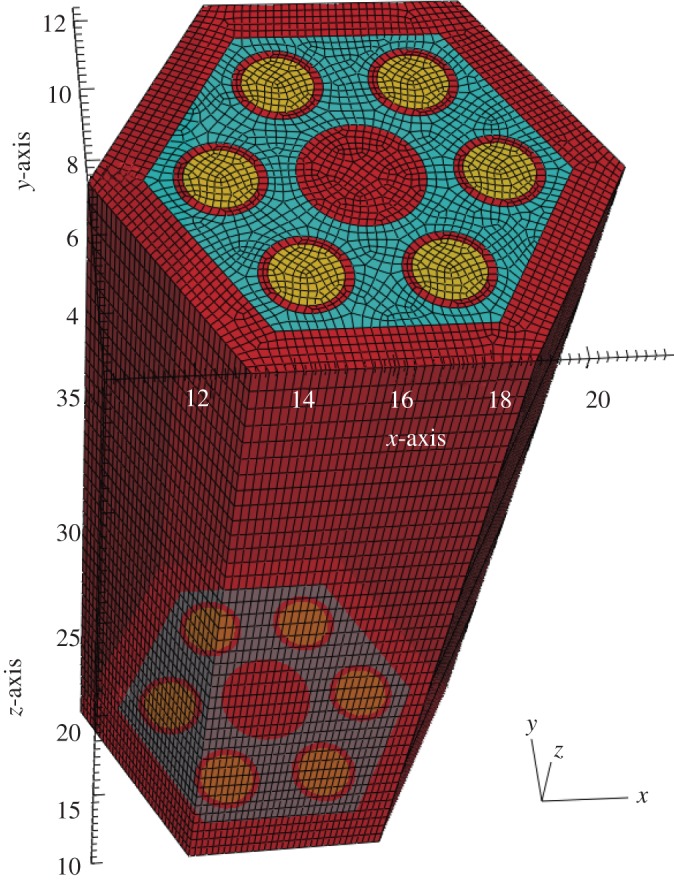
SAHEX problem geometry and sample mesh. (Online version in colour.)

The SAHEX problem geometry was designed to an outer duct wall that encloses six fuel pins and a control pin in the centre. Meshes with varying resolution were used for neutronics and thermal-hydraulics solvers to resolve the spatial scales in the model and the geometry consisting of fuel pins, cladding, control rod, steel can and sodium coolant. This model is carefully chosen to create a verification test case for solving realistic assemblies to be addressed later. The SAHEX geometry and a representative resolved physics mesh are shown in figure 4. The input data for the neutronics solver were generated *a priori* using the *MC*^2^ to obtain parametrized 9-group cross sections as a function of temperature and density. Since this is an isolated assembly model, a vacuum boundary (non-reentrant) condition is applied on the top and bottom and reflective boundary condition on all other outer surfaces of the neutronics model. The velocity and temperature boundary conditions are applied at the bottom surface of the model (Dirichlet for the fluid, Neumann for the solid) and outlet boundary conditions are applied at the top surface. In terms of the mesh resolution, the hydraulics solver uses a comparatively coarser mesh than neutronics, consistent with the use of quadratic elements and nature of spectral discretization.

In this section, the coupled results for the simplified single assembly are shown first and then the verification studies performed on this problem will be discussed.

#### (i) Initial conditions

**Figure 5.**
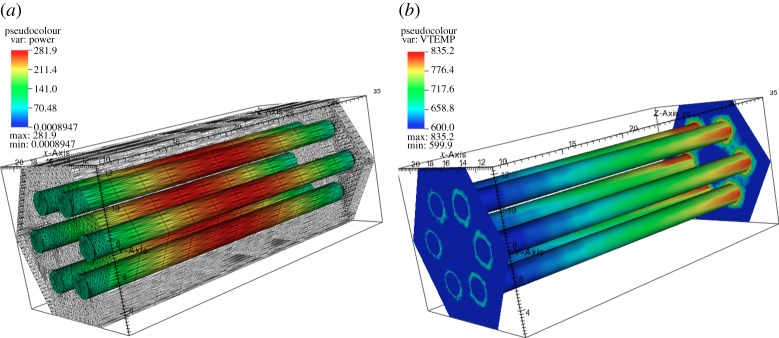
SS solution distributions. (*a*) Power profile (W) and (*b*) temperatures (K).

Following the procedure detailed in §3*a*, the SS solution fields for the individual physics were computed. The profile of the integral power based on the angular flux computed from solving equation (2.1) and the temperature profile from thermal-hydraulic solver are shown in figure 5. The decoupled profiles are physically meaningful and provide a good initial guess for coupled physics solver to obtain an SS solution. It is observed that the peak of the cosine shape of the power distribution shifts towards the inlet of the core due to lower material density at the top of the assembly, while the peak temperatures are observable near the outlet since the coolant temperature is monotonically increasing.

#### (ii) Solution verification

The convergence criteria for the neutronics and hydraulics solver were specified to be *rtol*_NE_=10^−7^ and *rtol*_TH_=10^−4^, respectively, in order to capture relative variations in the fine-scale scalar flux and temperature profiles. CouPE computes the rate of convergence and determines the stopping criteria for the coupled physics iteration. Every call to the thermal-hydraulic solver is preceded by a conservative solution projection of the power solution field computed in neutronics, which is used to recompute the volumetric power source in equation (2.7).

It is essential to rigorously verify spatial accuracy constraints in order to conserve the total energy specified for the problem configuration. We performed several successive refinements of the neutronics and hydraulics meshes, and quantified the error convergence in the solution projection and parallel data transfer algorithms. The results based on a reference mesh solution for the convergence of *k*_eff_ are tabulated in table 1. From the error convergence of uniform refinements of the mesh, it is evident that the numerical order of convergence of the coupled solver is nearly second order *O*(Δ*x*^2^) as expected, since the neutronics discretization is based on linear Lagrange basis functions. It is also imperative to note that the parallel performance of the solvers (on 32 processors) measured using the computational cost per Picard iteration (comprising the individual physics solvers and the two-way solution transfer of dependent fields) increases nearly linearly with the number of degrees of freedom.

**Table 1.** *k*_eff_ spatial convergence.

*N*_ele_	characteristic length	(computational time)/(Picard iteration) (s)	*k*_eff_	error (%)
7590	0.7144	44.64	0.67387473	0.42
54740	0.3573	63.39	0.67671801	0.114
437920	0.1791	84.27	0.67751902	0.0315
3503380	0.0895	162.93	0.67775429	reference

**Figure 6.**
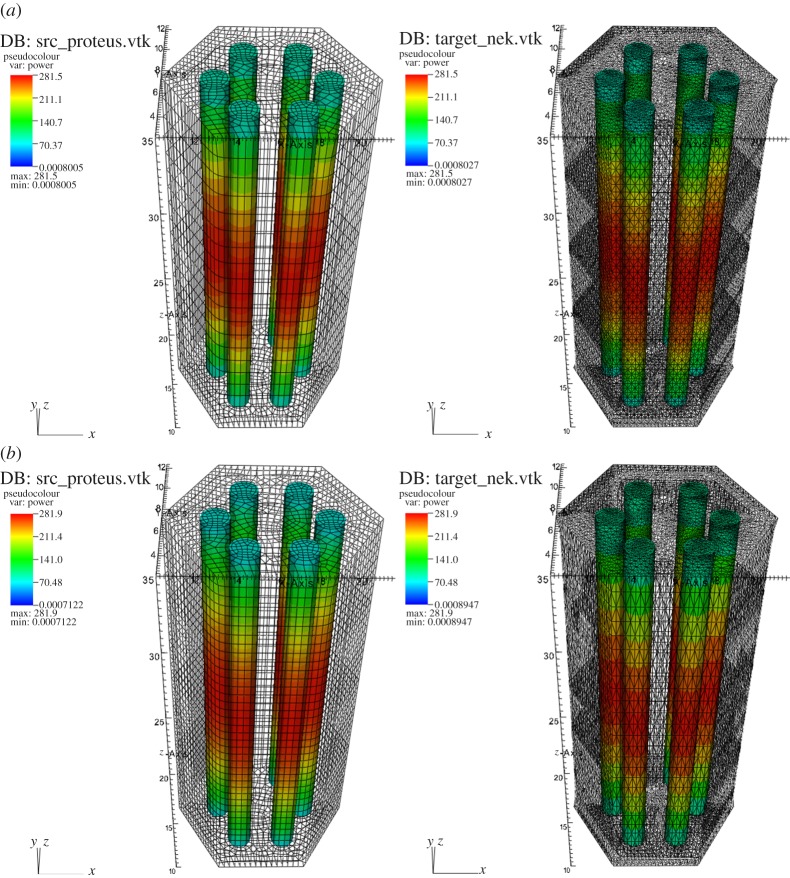
Power solution transferred from neutronics mesh to thermal-hydraulics. (*a*) Coarse resolution and (*b*) fine resolution.

The projected power solution at the end of the SS iteration from the source neutronics mesh to the target thermal-fluid mesh, after applying conservation prescriptions, is shown in figure 6*a*,*b* for a coarse and fine spatial resolution. It is evident from the scales and the integral of the profile distribution that the global energy is conserved during field transfers.

#### (iii) Coupled physics calculations

Once the ICs are converged, the loss-of-heat-sink simulation is initiated at *t*=*t*_0_, by updating the inlet boundary conditions as shown in equation (3.1) to increase inlet boundary temperature from 600 to 720 K during the transient. The total power in the assembly is specified by the user and power distributions are normalized accordingly.

**Figure 7.**
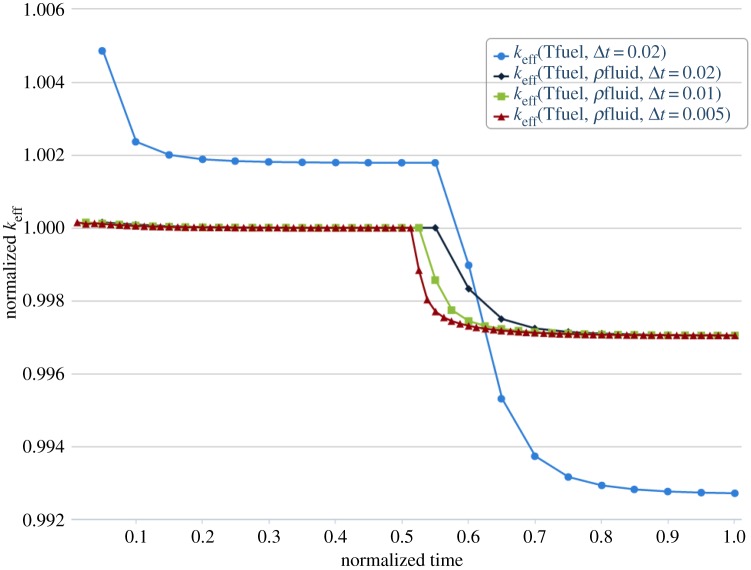
*k*_eff_ transient profile as a function of feedback and temporal resolution.

In all the cases, the number of subcycling steps performed in thermal-hydraulics was specified to resolve the transient change in temperature. Several transients have been performed to test for sensitivity of the coupled field solutions to different feedback effects. Figure 7 shows the change in *k*_eff_ as a function of normalized time for different types of coupled feedback effects optionally turned on. As the frequency of coupling is increased, the accuracy of the coupled physics solution improves since the computed criticality converges towards the reference. The flow time of the sodium through the assembly is 0.89 s (characteristic time scale), and the overall time steps are reduced consistently to resolve the spatial and temporal scales in successive simulations starting with Δ*t*_coarse_=0.02 s. The criticality constant *k*_eff_ rapidly converges as the number of time steps is halved while approaching the reference solution as *O*(Δ*t*). Note that feedbacks based on both temperature and density are necessary to show the complex nonlinear coupling between the neutronics and thermal-hydraulics physics for this test problem since the case where only Doppler feedback is considered shows larger sensitivity to the inlet temperature change. In other words, the density and Doppler expansion feedback are competing effects as validated from theory and experimental observations.

**Figure 8.**
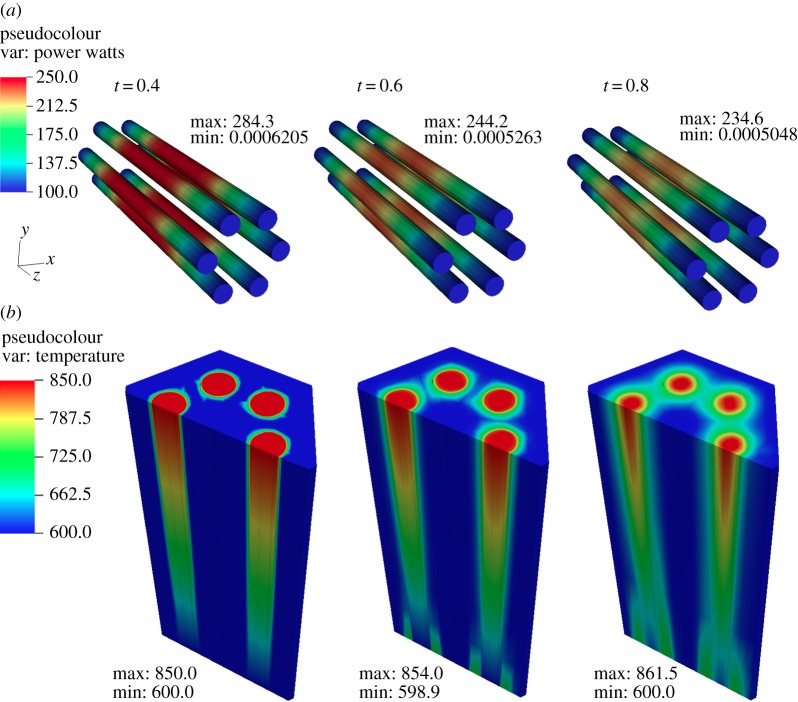
Transient evolution of coupled field profiles at the beginning, during and at the end of the perturbation. (*a*) Power distribution (W) and (*b*) temperature (K).

The total power decrease in the assembly as the transient progresses can be observed in figure 8*a*. The corresponding evolution of the temperature profiles is shown in figure 8*b*. Note that the significant change in power profile corresponding to only a minor penetration of the high temperature front within the domain indicates a very fast response (high sensitivity) to the boundary condition in the system.

The coupled physics simulation capability with the SHARP framework was tested on the SAHEX problem for a loss-of-heat-sink transient and the results obtained have been verified by spatial and temporal solution convergence studies. The sensitivity tests lead to important conclusions on:

(i) the time-step size necessary for this transient to maintain accuracy and
(ii) the importance of the inclusion of all types of feedback effects.

### (c) Realistic benchmark problem: XX09 assembly

Detailed specifications for the XX09 assembly can be found in [40,41]. Owing to the nature of the physics discretization (and similar to the SAHEX problem), the neutronics mesh is much more resolved than the corresponding fluid mesh in order to capture the heterogeneity in the geometry. The boundary conditions for the XX09 assembly problem for both neutronic and thermal-hydraulic solvers are similar to the SAHEX problem. The ICs are obtained by converging the PSS problem without any perturbations using the procedure described in §3*a*. The coupled loss-of-heat transient problem is simulated with this problem to observe spatio-temporal sensitivity and nonlinear feedback effects.

#### (i) Coupled solution results

**Figure 9.**
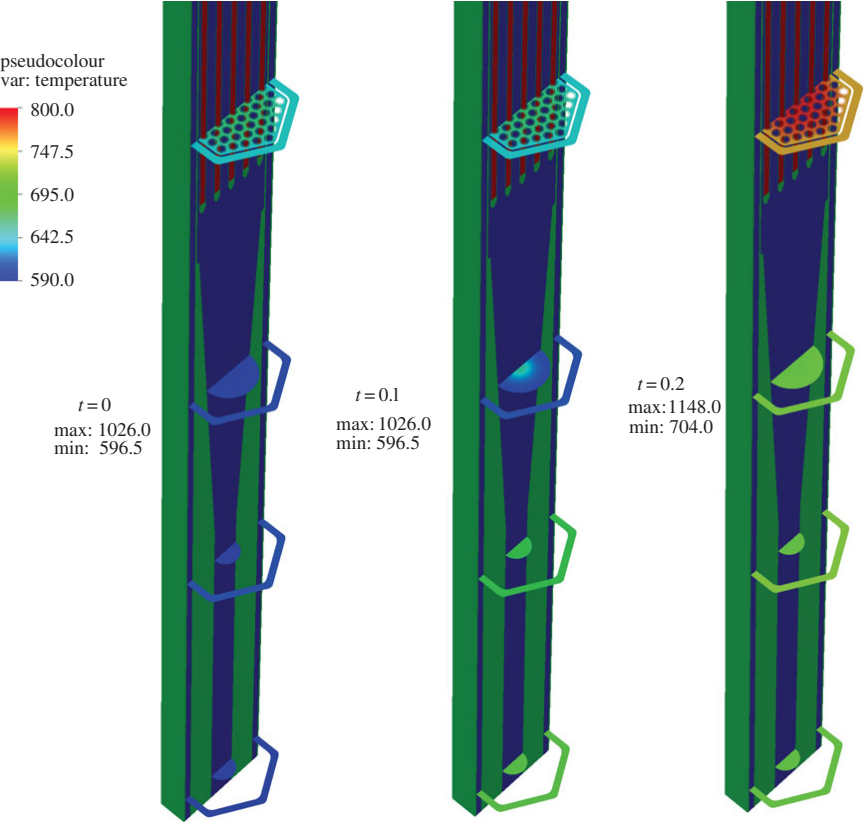
X–Z cross section of the Doppler temperature profile.

**Figure 10.**
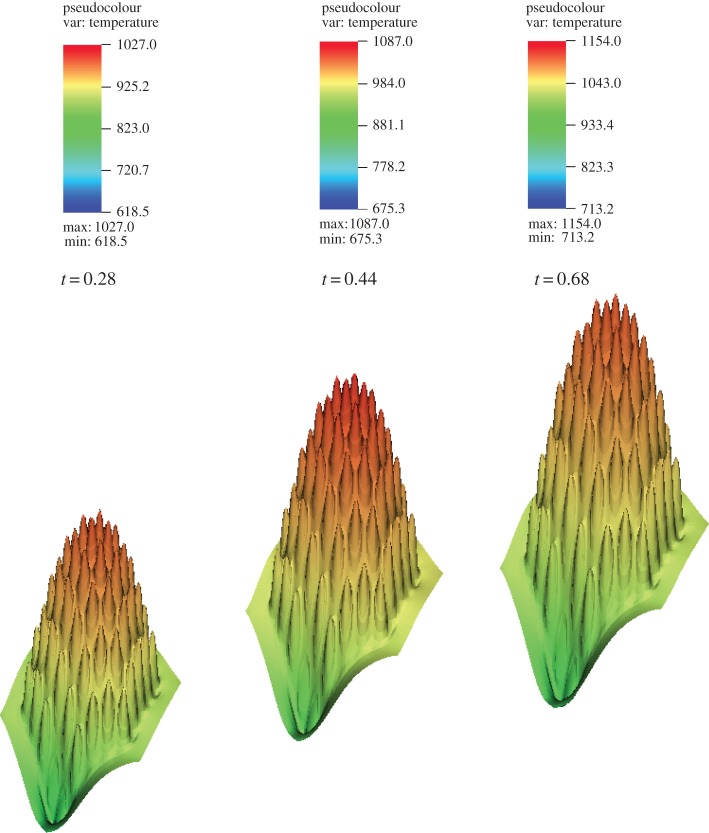
Temperature profile in the axial centre of the assembly.

The coupled physics results for the XX09 assembly problem use sub-cycled thermal-hydraulic solvers (5000 time steps, with a step size of Δ*t*=6×10^−6^). This step size was chosen to resolve the temporal evolution of the flow and temperature fields to approach an SS solution. A perturbation of the inlet temperature (with *α*=0.2 in equation (3.1) equivalent to a change of 120 K) is introduced and simulation is continued to convergence of a final SS condition. In the initial stage of the transient, a front with increased temperature slowly advances through the assembly as shown in figure 9. As expected, it was observed that the criticality *k*_eff_ reduces as the transient proceeds, owing to the negative feedback effect from the increase in fluid temperature flowing through the domain. The Doppler temperature increase is evident by observing the change in the profile at the axial centre of the assembly as shown in figure 10, which also contributes to decrease the sodium density, thereby adding to the negative feedback effects in the assembly as the temperature wave propagates.

**Figure 11.**
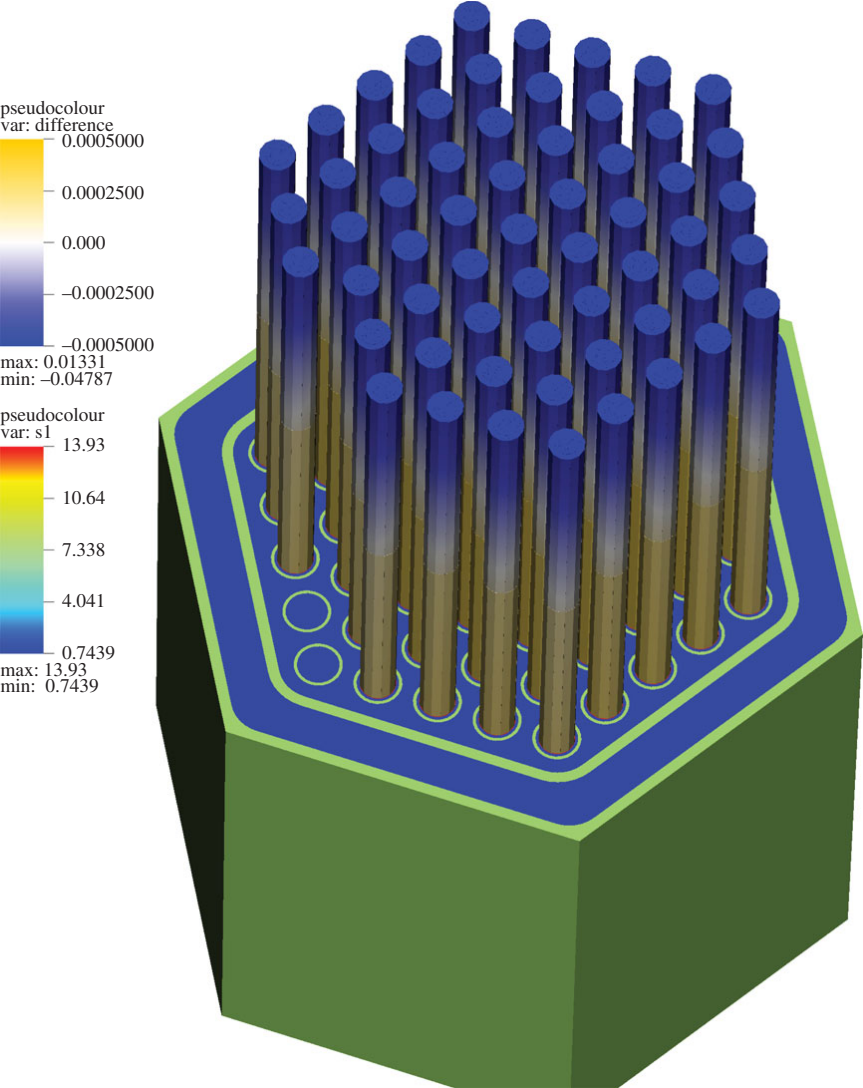
Shift in power distribution.

Figure 10 shows the relative change in power distribution between the final state of the transient and the initial SS result to quantify power sensitivity with respect to inlet temperature. In the second stage of the transient, the temperature increases in the fuel region producing the steepest change in reactivity and hence direct change in power distribution (figure 11). The results obtained from the simulation of XX09 assembly were verified to convergence towards reference solution by successive refinements to resolve the spatio-temporal scales in the physics. A high-resolution video of the loss-of-heat-sink transient for the XX09 assembly problem has been hosted externally [42]. It shows the detailed evolution of the temperature wave propagation along the axial length and the strong nonlinear feedback effects that quickly stabilize the power profile to a final equilibrium. Several representative meshes are also provided in order to aid readers to replicate and verify the nonlinear transient with their own coupled physics modelling codes.

## 4. Conclusion

The SHARP framework has been designed to tackle explicitly heterogeneous reactor assembly and full core geometry configurations and has been demonstrated to be convergent, and accurate in the implementation of the algorithms and the solver. The capability of the framework to flexibly choose different coupling schemes also makes it an important tool to determine the optimal solver method to resolve relevant scales for problems of interest. Using existing physics components expands the scope of SHARP to encompass different physics models with varying fidelity in equations, discretizations and requirements through a uniform abstraction layer. A structural mechanics solver, Diablo [43], is currently being integrated to analyse material deformation in the reactor due to large variations in the core temperature profile. Further investigations are also necessary to measure the overall parallel (MPI) performance of the tools for a multi-assembly problem on petascale architectures.

## Data accessibility

The supporting data for the problems described in the paper are hosted by a public git repository [42].

## References

[1] Keyes DE 2013 Multiphysics simulations: challenges and opportunities. Int. J. High Perform. Comput. Appl. 27, 4–83. (10.1177/1094342012468181)

[2] Larson J Jacob R Ong E 2005 The Model Coupling Toolkit: a new Fortran90 toolkit for building multiphysics parallel coupled models. Int. J. High Perform. Comput. Appl. 19, 277–292. (10.1177/1094342005056115)

[3] Siegel A Tautges T Caceres A Kaushik D Fischer P Palmiotti G Smith M Ragusa J 2007 Software design of SHARP. In Joint Int. Topical Meeting on Mathematics and Computations and Supercomputing in Nuclear Applications La Grange Park, IL: American Nuclear Society.

[4] Tautges TJ Kim H Caceres A Jain R 2011 Coupled multi-physics simulation frameworks for reactor simulation: a bottom-up approach. In Int. Conf. on Mathematics and Computational Methods Applied to Nuclear Science and Engineering (M&C 2011), Rio de Janiero, Brazil, 8–12 May 2011 La Grange Park, IL: American Nuclear Society.

[5] Bastian P Blatt M Engwer C Dedner A Kuttanikkad S Ohlberger M Sander O 2006 The distributed and unified numerics environment (DUNE).

[6] Gaston D Newman C Hansen G Lebrun-Grandie D 2009 MOOSE: a parallel computational framework for coupled systems of nonlinear equations. Nucl. Eng. Des. 239, 1768–1778. (10.1016/j.nucengdes.2009.05.021)

[7] Mahadevan VS 2010 High resolution numerical methods for coupled nonlinear multi-physics simulations with applications in reactor analysis. PhD dissertation College Station, TX, USA: Texas A&M University.

[8] Lowrie RB 2004 A comparison of implicit time integration methods for nonlinear relaxation and diffusion. J. Comput. Phys. 196, 566–590. (10.1016/j.jcp.2003.11.016)

[9] Mahadevan VS 2006 Nonlinearly consistent schemes for coupled problems in reactor analysis. Master's thesis College Station, TX, USA: Texas A&M University.

[10] Chauliac C Aragones J-M Bestion D Cacuci DG Crouzet N Weiss F-P Zimmermann MA 2011 NURESIM: a European simulation platform for nuclear reactor safety: multi-scale and multi-physics calculations, sensitivity and uncertainty analysis. Nucl. Eng. Des. 241, 3416–3426. (10.1016/j.nucengdes.2010.09.040)

[11] Xu Y Downar T Walls R Ivanov K Staudenmeier J March-Lueba J 2009 Application of TRACE/PARCS to BWR stability analysis. Ann. Nucl. Energy 36, 317–323. (10.1016/j.anucene.2008.12.022)

[12] Uspuras E Kaliatka A Bubelis E 2004 Validation of coupled neutronic/thermal-hydraulic code RELAP5-3D for RBMK-1500 reactor analysis application. Ann. Nucl. Energy 31, 1667–1708. (10.1016/j.anucene.2004.06.002)

[13] Seker V Thomas JW Downar TJ 2007 Reactor simulation with coupled Monte Carlo and computational fluid dynamics. In Proc. Int. Conf. on Emerging Nuclear Energy Systems (ICENES), Istanbul, Turkey, 3–8 June 2007.

[14] Griesheimer DP Gill DF Lane JW Aumiller DL 2008 An integrated thermal hydraulic feedback method for Monte Carlo reactor calculations. In Proc. Physor 2008 Conf., Interlaken, Switzerland, 14–19 September 2008. La Grange Park, IL: American Nuclear Society.

[15] Jareteg K Vinai P Demaziere C 2014 Fine-mesh deterministic modeling of PWR fuel assemblies: proof-of-principle of coupled neutronic/thermal-hydraulic calculations. Ann. Nucl. Energy 68, 247–256. (10.1016/j.anucene.2013.12.019)

[16] Strang G 1968 On the construction and comparison of difference schemes. SIAM J. Numer. Anal. 5, 506–517. (10.1137/0705041)

[17] Marchuk GI 1971 On the theory of the splitting-up method. In Proc. 2nd Symp. on Numerical Solution of Partial Differential Equations, pp. 469–500. New York, NY: Academic Press.

[18] Knoll DA Chacon L Margolin LG Mousseau VA 2003 On balanced approximations for time integration of multiple time scale systems. J. Comput. Phys. 185, 583–611. (10.1016/S0021-9991(03)00008-1)

[19] Ober CC Shadid JN 2004 Studies on the accuracy of time-integration methods for the radiation–diffusion equations. J. Comput. Phys. 195, 743–772. (10.1016/j.jcp.2003.10.036)

[20] Knoll DA Keyes DE 2004 Jacobian-free Newton–Krylov methods: a survey of approaches and applications. J. Comput. Phys. 193, 357–397. (10.1016/j.jcp.2003.08.010)

[21] Weniger EJ 2000 Prediction properties of Aitken's iterated Δ^2^ process, of Wynn's epsilon algorithm, and of Brezinski's iterated theta algorithm. J. Comput. Appl. Math. 122, 329–356. (10.1016/S0377-0427(00)00363-0)

[22] Tautges TJ Meyers R Merkley K Stimpson C Ernst C 2004 MOAB: a Mesh-Oriented database. Sandia National Laboratories, SAND2004-1592.

[23] Balay S Gropp WD McInnes LC Smith BF 1997 Efficient management of parallelism in object oriented numerical software libraries. In Modern software tools in scientific computing (eds Arge E Bruaset AM Langtangen HP), pp. 163–202. Basel, Switzerland: Birkhäuser Press.

[24] Tautges TJ Caceres A 2009 Scalable parallel solution coupling for multiphysics reactor simulation. J. Phys. Conf. Ser. 180, 012017 (10.1088/1742-6596/180/1/012017)

[25] Jiao X Heath MT 2004 Common-refinement-based data transfer between non-matching meshes in multiphysics simulations. Int. J. Numer. Methods Eng. 61, 2402–2427. (10.1002/nme.1147)

[26] Tautges TJ Kraftcheck J Bertram N Sachdeva V Magerlein J 2012 Mesh interface resolution and ghost exchange in a parallel mesh representation. In Proc. 26th IEEE Int. Parallel and Distributed Processing Symp. Workshops, Shanghai, China, 21–25 May 2012 1670–1679. (10.1109/IPDPSW.2012.208)

[27] Mahadevan VS Ragusa JC Mousseau VA 2012 A verification exercise in multiphysics simulations for coupled reactor physics calculations. Progr. Nucl. Energy 55, 12–32. (10.1016/j.pnucene.2011.10.013)

[28] Smith MA Rabiti C Palmiotti G Kaushik D Siegel A Smith B Tautges T Yang WS 2007 UNIC: development of a new reactor physics analysis tool. Trans. Am. Nucl. Soc. 97, 565–566.

[29] Lewis EE Miller WF 1984 Computational methods of neutron transport. New York, NY: John Wiley and Sons, Inc.

[30] Adams ML 1991 Even-parity finite-element transport methods in the diffusion limit. Progr. Nucl. Energy 25, 159–198. (10.1016/0149-1970(91)90007-C)

[31] Lee CH Yang WS 2012 MC^2-3^: multigroup cross section generation code for fast reactor analysis. Argonne National Laboratory, ANL/NE-11-41.

[32] Kaushik D Smith M Wollaber A Smith B Siegel A Yang WS 2009 Enabling high-fidelity neutron transport simulations on petascale architectures. In Proc. Conf. on High Performance Computing Networking, Storage and Analysis, pp. 67:1–67:12. New York, NY: ACM (10.1145/1654059.1654128)

[33] Mahadevan VS Smith MA 2011 Scalable multi-grid preconditioning techniques for the even-parity S_N_ solver in UNIC. In Int. Conf. on Mathematics and Computational Methods Applied to Nuclear Science and Engineering (M&C 2011), Rio de Janiero, Brazil, 8–12 May 2011 La Grange Park, IL: American Nuclear Society.

[34] Patera A 1984 A spectral element method for fluid dynamics: laminar flow in a channel expansion. J. Comput. Phys. 54, 468–488. (10.1016/0021-9991(84)90128-1)

[35] Maday Y Patera A 1989 Spectral element methods for the Navier–Stokes equations. In Stateof-the-art surveys in computational mechanics (eds Noor A Oden J), pp. 71–143. New York, NY: ASME.

[36] Fischer P Patera A 1991 Parallel spectral element of the Stokes problem. J. Comput. Phys. 92, 380–421. (10.1016/0021-9991(91)90216-8)

[37] Fischer P 1997 An overlapping Schwarz method for spectral element solution of the incompressible Navier–Stokes equations. J. Comput. Phys. 133, 84–101. (10.1006/jcph.1997.5651)

[38] Tomboulides A Israeli M Karniadakis G 1989 Efficient removal of boundary-divergence errors in time-splitting methods. J. Sci. Comput. 4, 291–308. (10.1007/BF01061059)

[39] Tomboulides A Lee J Orszag S 1997 Numerical simulation of low Mach number reactive flows. J. Sci. Comput. 12, 139–167. (10.1023/A:1025669715376)

[40] Poloncsik J Filewicz EC Kamis GJ Natoce JT 1982 The experimental breeder reactor II (EBR-II) instrumented subassemblies, INSAT XX09 and INSAT XX10. In Proc. Conf. on Fast, Thermal and Fusion Reactor Experiments, Salt Lake City, UT, 12–15 April 1982, pp. 1-276–1-287. La Grange Park, IL: American Nuclear Society.

[41] Tautges TJ Fischer P Grindeanu I Jain R Mahadevan V Obabko A Smith MA Merzari E Ferencz RM 2013 SHARP assembly-scale multiphysics demonstration simulations. Argonne National Laboratory, technical report ANL/NE-13/9.

[42] Mahadevan VS Merzari E Tautges T Jain R Obabko A Smith M Fischer P 2014 A public git repository that hosts all the supporting data for the problems described in the current paper published in. Phil. Trans. R. Soc. A. See https://bitbucket.org/vijaysm/rsocpaper2014.

[43] Parsons I Solberg J Ferencz R Havstad M Hodge N Wemhoff A 2007 Diablo user manual. Lawrence Livermore National Laboratory, UCRL-SM-234927.

